# Mechanistic insights into *Calotropis procera* extract and green-silver nanoparticles as therapeutic agents in murine schistosomiasis: Targeting BAX/Bcl-2 and oxidative stress

**DOI:** 10.5455/javar.2025.1992

**Published:** 2025-12-29

**Authors:** Zeyad K. Hamdan, Muzayyan Fadhly Namik, Noor Adnan Mahmood, Mohammad I. Soliman, Mohammed A. Abdel-Rasol, Ahmed H. Nigm

**Affiliations:** 1 Department of Biology, College of Education for Pure Science, Tikrit University, Tikrit, Iraq; 2 Department of Zoology, Faculty of Science, Ain Shams University, Cairo, Egypt ahmednigm@sci.asu.edu.eg (A.H.N.)

**Keywords:** Liver functions, Apoptotic markers, Antioxidant enzymes, Sodom apple, Schistosoma mansoni, Biogenic nanoparticles

## Abstract

Objective: This study aims to make a comparative evaluation of the therapeutic efficacies of the Calotropis extract (CPE) and green silver nanoparticles (GSNPs) compared to praziquantel (PZQ) in treating mice infected with schistosomiasis, with a comprehensive assessment of host physio-logical responses. Materials and Methods: The CpE-fabricated silver nanoparticles were characterized (UV-vis spectroscopy, transmission electron microscopy, and Fourier transform infrared). Five experimen-tal groups were conducted: uninfected, infected untreated, PZQ-, CpE-, and GSNP-treated. The parasitological, biochemical, histopathological, and ultrastructural approaches were employed in evaluating each treatment protocol. Results: It was found that the reduction of the worm burden and oogram count was significantly enhanced by both CpE and GSNPs, yet PZQ showed better antiparasitic effects. The antioxidant activity of GSNPs was more efficient, and they significantly restored hepatic (glutathione) as well as reduced (malondialdehyde) and (nitric oxide) levels. In addition, both CpE and GSNPs regulated apoptotic markers by inhibiting Bcl2-cell-associated X protein and enhancing B-cell lymphoma protein 2 expression, thereby inhibiting hepatocyte apoptosis. Histopathological examination revealed a reduction in granuloma size and hepatic fibrosis, along with an improved hepatoprotective potential of GSNPs. Biochemical analysis revealed increased activity of liver enzymes (alanine transaminase, aspartate transaminase, and gamma-glutamyl transferase) as well as markers of renal function (urea and creatinine), indicating they also had a systemic protective role. All treatments resulted in some ultrastructural alterations in worms. Conclusion: The normalized parasitological and physiological parameters were utilized in develop-ing a novel mathematical framework that facilitates more objective assessments for treatment pro-tocols. Although PZQ showed the highest efficacy against the parasite (total efficacy score: 71.8), the plant extract showed the highest efficacy in anti-fibrotic and anti-apoptotic properties (total efficacy score: 61.6). However, GSNPs showed balanced therapeutic benefits (total efficacy score: 51.1). This study indicated that comparison based on both the host physiological parameters and those of the parasite is more comprehensive for optimal anti-schistosomal treatment selection. This study also confirmed that treatments derived from plant sources may have complementary benefits over traditional chemotherapy through metabolic pathways involved in the host protective responses.

## Introduction

Schistosomiasis affects approximately 600 million people living in tropical and subtropical regions each year, mak-ing it the second most common disease in the world after malaria [1]. This situation calls for research into anti-schis-tosomiasis treatments, especially those derived from plant sources [2]. *Calotropis procera* is a medicinal plant that has been demonstrated to possess anticarcinogenic [3], antibacterial [4], anthelmintic [5], and antioxidant [6] prop-erties. In previous studies on schistosomiasis, it was found that the extract of this plant has anti-inflammatory effects in addition to destructive activities on the tegument of worms [5],[7].

Metal-fabricated nanoparticles, including those made of silver and gold, have emerged as potential alternatives for treating *Schistosoma* parasites due to their rapid and cost-effective availability [8]. However, their minute size allows them to infiltrate even the body's small blood ves-sels, concentrating their therapeutic effects on the para-sites while minimizing their adverse effects. Additionally, research has shown that silver nanoparticles can effectively combat the *Schistosoma* parasite [9]. Silver nanoparticles were chosen due to their antibacterial effects [10] and high biocompatibility, especially when formed by plant extracts [11]. Additionally, silver nanoparticles are an excellent and inexpensive strategy [12]. The antiparasitic activity of sil-ver nanoparticles is attributed to the release of free silver ions, which affects both the parasite and the host, inducing oxidative stress [8]. Green nanotechnology plays a broader and deeper role, as recent studies have demonstrated its contributions to diverse biomedical and non-biomedical applications [13],[14]. Several studies have evaluated the antischistosomiasis effects of *Calotropis*-fabricated silver nanoparticles [10],[15].

*Calotropis procera* (Aiton) W.T. Aiton (family: Apocynaceae) is a perennial shrub widely distributed in desert regions. This plant is rich in bioactive secondary metabolites, including cardenolides, flavonoids, and phe-nolic compounds, which have demonstrated significant antioxidant, anti-inflammatory, anthelmintic, antibacte-rial, and anticancer properties [16]. The antischistosomal effect of *Calotropis*-mediated silver nanoparticles was con-firmed in a previous study [5]. Therefore, the multifaceted pharmacological profile of *C. procera*, combined with the advantages conferred by plant-mediated nanoparticle fab-rication, provides a rational and promising basis for its selection in this comparative investigation.

Most studies focus on the direct effects of treatments on parasite morphology and physiology, neglecting host responses. Yet drug effects in infected mice can differ markedly from those in healthy animals due to parasite-in-duced changes in the host environment. This necessitates performing simultaneous physiological examinations on the infected murine systems and reducing reliance on data derived from research that applied the same drug on cell lines (*in vitro*) or healthy animals.

In this study, a comparative evaluation of three dif-ferent treatment protocols against *Schistosoma mansoni* was conducted [intraperitoneal injection with *Calotropis*-mediated silver nanoparticles, oral administration of *Calotropis* extract (CpE), and the praziquantel (PZQ) drug]. This comparison included recording changes in some physiological indicators of the murine system, in addition to the anti-schistosomiasis effects. The applied physiolog-ical parameters were the liver enzymes in serum, includ-ing aspartate transaminase (AST), alanine transaminase (ALT), and gamma-glutamyl transferase (*y*-GT). Besides, apoptotic Bcl2-cell-associated X protein (BAX), and B-cell lymphoma protein 2 (Bcl2), antioxidant (superoxide dis-mutase (SOD), and catalase (CAT), and hepatic oxidative stress markers [(reduced glutathione (GSH), malondial-dehyde (MDA), and nitric oxide (NO)] were measured in liver tissues. The applied parasitological criteria included the recovery of adult worms, oogram pattern, egg count in both the liver and intestine, and the size and number of granulomas, as well as a quick view of the ultrastructural modifications in the adult worm tegument.

## Materials and Methods

### Ethical approval

All animal procedures and experimental protocols con-cerning this work were approved by the Ain Shams University Research Ethics Committee (ASU-REC), Egypt (Code: ASU-SCI/ZOOL/2025/3/3).

### Chemicals

The plant under investigation was collected from the des-ert areas in eastern Cairo, specifically near the Joseph Tito Highway, 30&deg;08'20.3"N 31&deg;25'08.7"E. The plant was identi-fied by a colleague (Dr. Mohammed Abdel Fattah) from the Botany Department, and a voucher specimen (giant milk-weed-4-17) was deposited at the Department of Botany, Faculty of Science, Ain Shams University (Cairo, Egypt). PZQ (Distocide&reg;) was purchased from EIPICO, El-Asher Men Ramadan (Cairo, Egypt). Cremophore&reg; EL was obtained from Sigma-Aldrich&reg; Chemical Company (Germany).

### Preparation of aqueous CpE

The fresh stems of *C. procera* were separated, washed to remove impurities, dried in a shadowed area, and then ground with a mortar apparatus. The distilled water (500 ml) was mixed with the plant powder (300 gm) in a clean glass beaker. The mixture was heated to boiling point (30 min) and then cooled at room temperature. It was sub-sequently filtered through muslin cloth and Whatman&reg; qualitative filter paper, Grade 1. The filtrate was centri-fuged at 2,500 rpm for 15 min to remove any remaining particles, resulting in a clear supernatant that was subse-quently stored in a dark container at 4&deg;C for future use.

### Preparation and characterization of green silver nanoparti-cles (GSNPs)

GSNPs were synthesized by adding 15 ml of *C. procera* extract dropwise to a 4 mM silver nitrate solution (0.7 gm AgNO_3_ in 1 L deionized water), with continuous stirring of the mixture at 60&deg;C-70&deg;C by a thermal magnetic stirrer (FineLab&reg; Scientific, Hong Kong). The color of the mixture changes from pale yellow to dark brown after 4 h, indicat-ing nanoparticle formation. After 24 h, the nanoparticles were collected by centrifugation at 15,000 rpm for 5 min, washed 3 times with deionized water, dried, and stored at 4&deg;C [17]. The structure of GSNPs was confirmed using a UV-vis spectrophotometer (Shimadzu&reg; UV-visible 1,800 Spectrophotometer, Japan). The sample was diluted appro-priately to avoid saturation at the plasmon resonance peak. The samples' absorption spectrum was measured between 200 and 1,000 nm with the following settings: double-beam, baseline correction using distilled water, and scan speed of 240 nm/min. To determine the size distribu-tion of the resultant GSNPs, dynamic light scattering (DLS) (Particle Sizing Systems, NICOMP&trade; 380NLS, Santa Barbara, CA) was employed. Silver nanoparticle suspensions were diluted in ultrapure water to an optimal concentration and filtered to remove dust or large aggregates. Instrumental settings included wavelength 633 nm (He-Ne laser), detec-tion angle: backscatter angles, 3-5 runs of 10-30 sec per run, and 25&deg;C.

The surface adsorption of functional groups on green nanoparticles was studied using Fourier transform infra-red (FTIR) spectrum measurements (Jasco&reg; FT/IR 6100, Japan). The sample was deposited as a thin film on a glass substrate, and then the measurements were carried out over the mid-infrared region, typically from 4,000 to 400 cm^&minus;1^ with the following instrumental settings: res-olution of 4 cm^&minus;1^, 32 to 64 scans, atmosphere: nitrogen. Furthermore, the form and particle size of GSNPs were determined with transmission electron microscopy (TEM) on a Philips/FEI Inspect-F (Philips/FEI&reg;, USA). The nano-suspension was diluted appropriately in distilled water, and a drop (5-10 &micro;l) was placed on a carbon-coated cop-per TEM grid, which was air-dried before imaging. The instrumental settings: 100 to 120 kV, Bright field, thermi-onic emission gun, and magnification range from 150,000x to 250,000x [18].

### Experimental animals and infection

Throughout the study, male Swiss albino mice weighing approximately 20 &plusmn; 5 gm at the start of the experiment were employed. The animals were acquired from the Theodor Bilharz Research Institute, Schistosome Biology Supply Centre (SBSC), in Giza, Egypt, and they were kept in appropriate cages for a week to allow them to be acclimatized to the laboratory environment (12-h dark/ light cycle with free access to fresh water and a standard commercial pellet diet). The fresh cercariae of *S. mansoni* were also acquired from SBSC for infection. The mice were experimentally infected by fresh *S. mansoni* cercariae (70 &plusmn; 10/mouse) via subcutaneous injection [5].

### Experimental design

Twenty-five male mice were divided into five groups for this study (*n* = 5 per group). Group I was the negative control (non-infected), whereas groups II through V were infected with *Schistosoma*. Group II served as the positive control (untreated) group. Group III orally received PZQ, prepared in 2% (v/v) Cremophor EL, at a dose of 500 mg/kg for 2 consecutive days [8],[13]. Group IV was orally administered with *C. procera* extract at 200 mg/kg for 15 days [19]. Group V was treated intraperitoneally with a modified dose of GSNPs, 200 mg/kg, administered in six doses over a 2-week period [5]. All treatments were applied in the 6th week post-infection. GSNP suspension was prepared using phosphate buffer vehicles.

### Blood sampling and tissue extraction

Animals received isoflurane anesthesia and were sacri-ficed at the end of the experiment. After the blood sam-ples were collected, they were coagulated in sterile test tubes. The serum was then separated by centrifuging the blood samples at 4,000 rpm for 20 min at 4&deg;C, and subse-quently aliquoted and stored at -80&deg;C for further analysis. Immediately after sacrifice, the livers were perfused (Porto-mesenteric perfusion technique) using cold isotonic citrate saline, which was forced through the hepatic portal vein to collect all recovered worms from the hepatic and Porto-mesenteric veins. The livers were then dissected, weighed, and kept in aluminum foil at -80&deg;C. For further prepara-tion, liver tissues were washed with cold isotonic saline, and 1 gm of liver was homogenized in 10 ml of cold 0.1 M potassium phosphate buffer (pH 7.3). The homogenate was centrifuged at 10,000 rpm for 20 min at 4&deg;C; the super-natant was collected and stored at -80&deg;C for subsequent assays, with an aliquot retained for protein determination.

### Parasitological parameters

#### Total worm burden (TWB)

After 2 weeks of treatment, mature schistosomes were recovered from the portal mesenteric veins according to the protocol [20].

#### Oogram count

The proportions of various egg developmental stages (immature, mature, or dead) in the intestines were exam-ined as described in [21]. The number of eggs at each stage was divided by the overall egg count corresponding to a given weight of intestinal tissue.

#### Tissue egg loads of the hepatic/intestinal tissues

The liver and intestines were sampled (two samples per organ), weighed, and then frozen at -20&deg;C. A potassium hydroxide (KOH) solution (4%) was used to digest the samples. The total number of eggs in the liver and intes-tines was calculated according to [22]. Briefly, the tissue egg load was calculated as follows: the mean of the egg number in homogenized tissue = the mean number of eggs in the microscopically examined sample was multiplied by the volume of digested tissue, and then it was divided by the volume of the microscopically examined sample. Then, the mean number of ova per gram in the infected organ was computed as the sum of the number of ova/gm in two representative samples and dividing the result by 2.

#### Histopathological studies

The isolated pieces of liver were rinsed in 10% formal saline and subsequently fixed in a 10% neutral buffered formalin solution. For sectioning, the specimens were rinsed with distilled water, dehydrated, and embedded in paraffin wax. Five sections (5-&micro;m thickness) were placed on a clean glass slide for each liver sample. The transparencies were dep-araffinized and stained with trichrome, hematoxylin, and eosin stains. The hepatic lesion containing a central single ovum was employed to achieve microscopical examination of specimens [23]. Furthermore, the mean diameter of the selected granuloma was determined by obtaining the max-imal diameter and its perpendicular one [24].

#### Transmission electron microscopy

The recovered worms were washed with physiological saline and then fixed for 2 h in 3% glutaraldehyde and 0.1 M phosphate buffer (PH 7.4). Then, the specimens were subjected to post-fixation in 1% osmium tetroxide for 1 h, followed by dehydration and embedding in epoxy resin. The semithin sections were stained with methylene blue and examined by light microscope (Olympus&reg; CX23 bin-ocular microscope, Japan), and then the ultrathin sections were stained with uranyl acetate/lead citrate and exam-ined by TEM (Philips/FEI Inspect-F, Philips/FEI&reg;, USA) in the regional center for mycology and biotechnology, Al-Azhar University, Cairo, Egypt.

#### Biochemical assays

Oxidative stress and antioxidant markers were assessed in liver tissue homogenates. The parameters investi-gated included MDA, NO, SOD, CAT, and reduced GSH. The activities of these markers were determined according to [25]-[29], respectively. Additionally, the apoptotic markers (BAX and Bcl-2) were measured in the homogenates of liver using ELISA kits: BAX (Catalog No: SEB631R, Cloud-Clone&reg; Corp., USA) and Bcl-2 (Catalog No: E-EL-R0648, Elabscience&reg; Biotechnology Inc, USA), following the man-ufacturer's instructions. The liver enzymes (AST, ALT, and *y*-GT) as well as kidney function indicators (urea and cre-atinine), were also analyzed.

#### Statistical analysis

The data were given as *mean* &plusmn; *standard error of the mean* (SEM). To evaluate statistically significant differences among experimental groups, One-way analysis of variance (ANOVA) was implemented, followed by Tukey's multi-ple comparison test. *P*-values were considered significant when *p* < 0.05. SPSS software, version 24, was used for data analysis, Chicago, IL.

## Results

### Characterization of GSNPs

**Figure 1:**
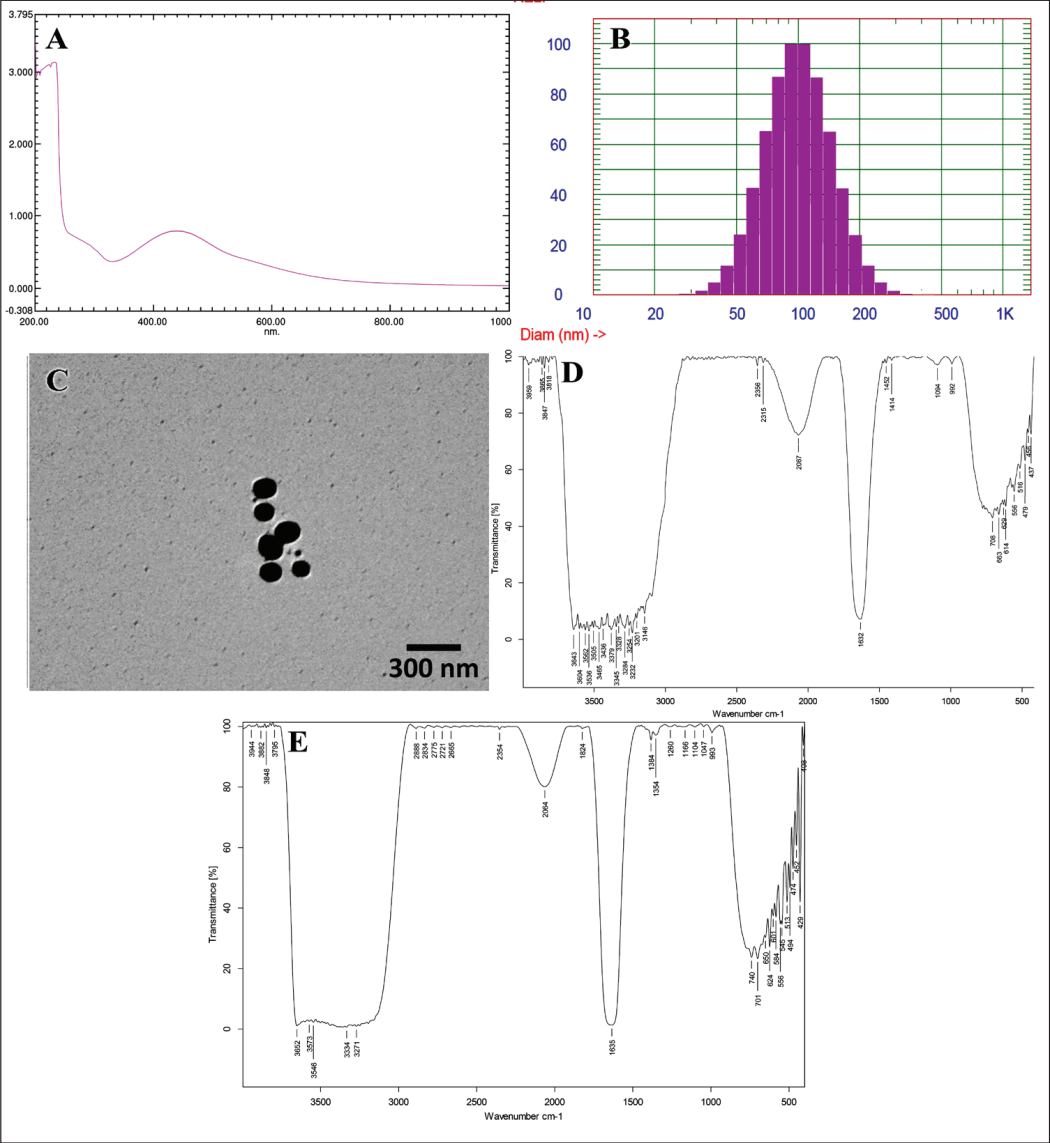
Characterization of GSNPs. A: UV-vis spectra, B: DSL analysis, C: TEM profiles, D: FTIR spectra of plant extract, E: FTIR spectra of GSNPs.

UV-visible spectroscopy was employed to verify the forma-tion of green nanoparticles. The maximum spectral absorp-tion was observed at 426 nm, which is indicative of silver nanoparticles (Fig. 1A). In contrast, the particle size analy-sis conducted using DLS demonstrated the formation of a polydisperse population of nanoparticles within a 110 nm range (Fig. 1B). The transmission microscopical examina-tion showed that synthesized GSNPs have a relatively uni-form spherical pattern with an average diameter of 108.5 &plusmn; 32 nm (Fig. 1C). The infrared spectra (FTIR) of both the plant extract (CpE) and GSNPs showed that the bands of the absorption of both the extract and GSNPs were more or less identical; that means the presence of capping agents over the surfaces of the formed silver nanoparticles (Fig. 1D, E).

Figure 1 Characterization of GSNPs. A: UV-vis spectra, B: DSL analysis, C: TEM profiles, D: FTIR spectra of plant extract, E: FTIR spectra of GSNPs.

Table 1

Results are expressed as the Mean &plusmn; SEM. ^a, b^ indicate significant differences versus infected and PZQ groups, respectively at *p*-value < 0.05.

### Parasitological parameters

#### The TWB

It was observed that none of the treated groups recov-ered any female worms, which represented a statistically significant difference (*p* < 0.05) from the infected control group. The most significant reduction in worm burden was observed in the PZQ-treated group (90.6%), with statis-tical differences (*p* < 0.05), followed by the plant extract-treated group (60.47%) (Table 1).

#### The oogram pattern

The PZQ-treated group exhibited the greatest percentage of dead eggs (94%, *p*-value < 0.05). Furthermore, the per-centage of dead embryos in both groups treated with either plant extract or green nanoparticles is not significantly dif-ferent (16.4%and 11.8%, respectively). Conversely, the PZQ-treated group did not exhibit any immature eggs (Table 2).

#### Tissue egg load

In comparison to the infected group, the PZQ-treated group exhibited the maximum reduction percentage of total egg count in tissue (92.7%), followed by the plant extract-treated group (29.39%) (Table 3). It was observed that the number of ova in the liver was sig-nificantly lower in the CpE group than in the intestine. Conversely, the GSNPs or CpE-treated groups did not exhibit significant reductions in tissue egg load of the intestine.

Table 2

Results are expressed as the Mean &plusmn; SEM. ^a, b, c^ indicate significant differences versus infected, PZQ, and CpE groups, respectively at *p*-value < 0.05.

Table 3

Results are expressed as the Mean &plusmn; SEM. ^a, b^ indicate significant differences versus infected and PZQ groups, respectively at *p*-value < 0.05.

### Histopathological changes in liver tissues

**Figure 2:**
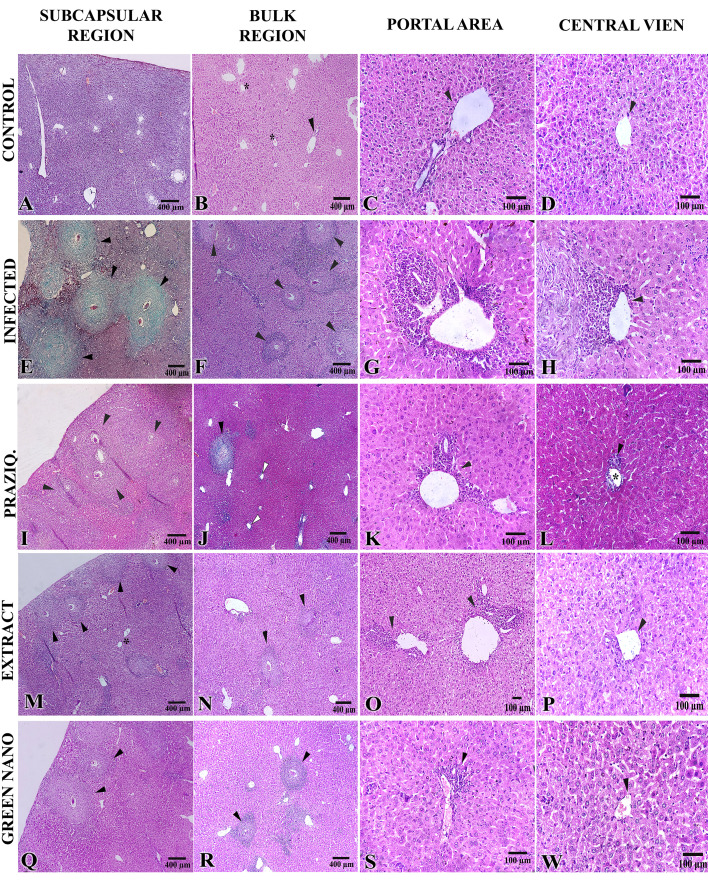
Sections of the liver show the histological features in the experimental groups. A&ndash;D: negative control group. E&ndash;H The infected group. I&ndash;L: PZQ-treated group. M&ndash;P: Plant extract group. Q&ndash;W: GSNPs-treated group.

The negative group showed normal liver architecture (Fig. 2A), including the reticular architecture of hepato-cytes (Fig. 2B), healthy portal zones (Fig. 2C), and normal central veins (Fig. 2D), with no inflammatory infiltra-tion observed. In the infected group, schistosomiasis-re-lated histopathological aspects were observed, including numerous scattered fibrocellular granulomas (Fig. 2E), particularly in the subcapsular hepatic regions (Fig. 2F), with inflammatory infiltrates surrounding the dilated por-tal spaces (Fig. 2G). In addition, inflammatory infiltrations were observed around the slightly dilated central vein (Zone III) (Fig. 2H).

In comparison to the infected group, the PZQ-treated group exhibited the maximum reduction in granuloma size and count (46.3% and 74.8%, respectively) (Table 4; Fig. 2I, G). Lower reductions in the granuloma size/count (19.3%, 28%, respectively) were observed in the CpE-treated group (Fig. 2M, N).

On the other hand, the GSNPs-treated group exhib-ited only a significant change in granuloma count (16%) (Fig. 2O, R). Concerning the histopathological changes, the GSNPs-treated group showed a better healthy profile in the portal spaces and centrilobular zone (Fig. 2S, W). In contrast, the PZQ and, to a lesser extent, CpE-treated groups exhibited inflammatory infiltrates in these areas, along with dilatations in both portal and central veins (Fig. 2K, L, O, P).

**Figure 3:**
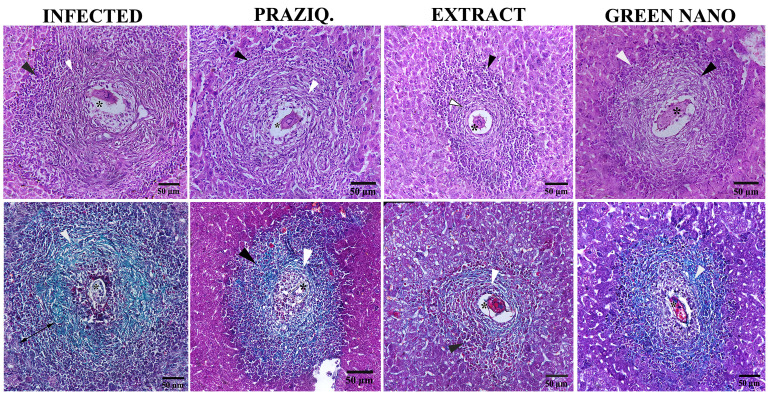
High-power magnifications for granulomas showing the size and the histologicalfeatures of granulomas, the upper row for sections stained with H&E, and the lower one stained with trichrome stain (white arrowheads represent the fibrous layers, black arrowheads represent inflammatory cells, and asterisks represent the ova).

In the infected group, most of the granulomas were fibrocellular (Table 4), composed of inner thick fibrous layers and outermost layers of inflammatory cells (Fig. 3). The percentage of fibrocellular granulomas was greatly increased in the PZQ-treated group (96.3%), followed by that of the plant extract-treated one (90.6%) (Table 4). It was noticed that the fibrous content of the granuloma (in hepatic sections stained with trichrome) was generally the lowest in plant extract-treated mice (Fig. 3).

Figure 2. Sections of the liver show the histological features in the experimental groups. A-D: negative control group. E-H: The infected group. I-L: PZQ-treated group. M-P: Plant extract group. Q-W: GSNPs-treated group.

Table 4

Results are expressed as the Mean &plusmn; SEM. ^a, b, c^ indicate significant differences vs. infected, PZQ, and CpE groups, respectively at *p*-value < 0.05.

Figure 3. High-power magnifications for granulomas showing the size and the histological features of granulomas, the upper row for sections stained with H&E, and the lower one stained with trichrome stain (white arrowheads represent the fibrous layers, black arrowheads represent inflammatory cells, and asterisks represent the ova).

### TEM examination of recovered adult worms

**Figure 4:**
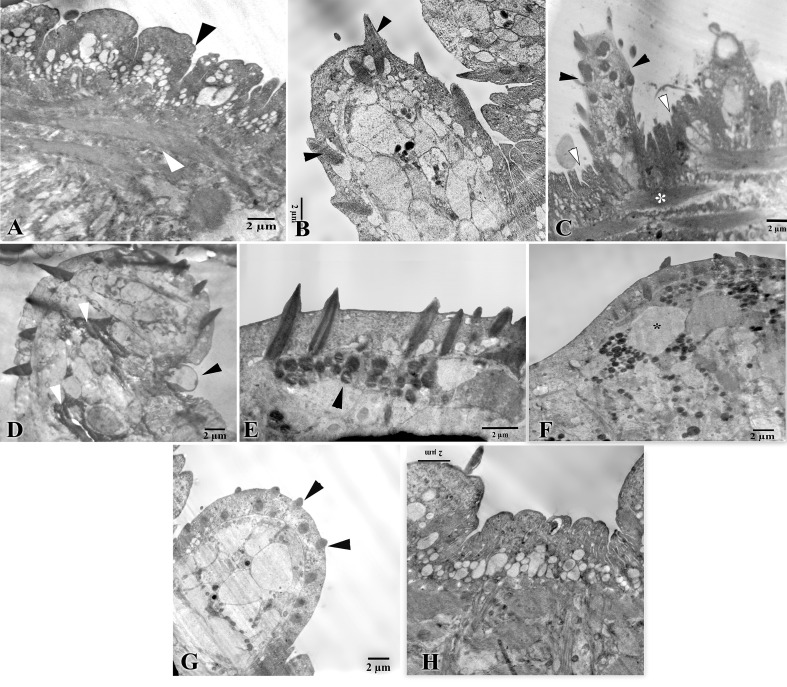
TEM of the tegumental surface of an S. mansoni male. A&ndash;B: Control group; A, the tegument shows a healthy appearance, including the highly convoluted outer surface (black arrowhead) and the prominent musculature (white arrowhead). B, a tubercle with prominent sharp spines (black arrowheads). C&ndash;F: Extract-treated group; C, the tegumental surface is highly folded (white arrowheads) with the presence of an obvious collapse in tubercles (black arrowheads). D, thetegument shows a bleb in the base of a tubercle (black arrowhead), besides some dead parenchymal cells (white arrowheads). E, Abnormal aggregations of mitochondria in a parenchymal region under the tegument, which contains spines (black arrowhead). F, large vacuoles in the parenchymal region (asterisk). G&ndash;H: G, Green nanoparticle-treated group: the normal-sized tubercles with blunt, short, reduced spines (black arrowheads). H, The tegumental area with a normal profile.

The potential surface modifications in the tegument of male worms were briefly examined in this study. In the untreated infected group, the male tegument was highly convoluted, with numerous large channels that were open on the surface; moreover, tubular invaginations formed from the basal lamina into the tegument. The muscula-ture was continuous with the normal healthy architecture and prominent spiny tubercles (Fig. 4A, B). In the extract-treated group, a highly folded tegumental surface (in the dorsal side of the worm) was observed with the presence of an obvious collapse in tubercles and discontinuity during the subtegumental musculature (Fig. 4C). Besides, the tegument showed some blebs around tubercles accom-panied by dead parenchymal cells (Fig. 4D). Characteristic abnormal aggregations of mitochondria were observed, especially in tegumental regions under spines with large vacuoles in parenchyma (Fig. 4E, F). In the GSNP-treated group, the tegument exhibited a minimum state of patho-logical changes where the normal-sized tubercles had blunted, short, reduced spines (Fig. 4G); the remaining ultrastructural features of the tegument were normal (Fig. 4H).

Figure 4. TEM of the tegumental surface of an S. mansoni male. A-B: Control group; A, the tegument shows a healthy appearance, including the highly convoluted outer surface (black arrowhead) and the prominent musculature (white arrowhead). B, a tubercle with prominent sharp spines (black arrowheads). C-F: Extract-treated group; C, the tegumental surface is highly folded (white arrowheads) with the presence of an obvious collapse in tubercles (black arrowheads). D, the tegument shows a bleb in the base of a tubercle (black arrowhead), besides some dead parenchymal cells (white arrowheads). E, Abnormal aggregations of mitochondria in a parenchymal region under the tegument, which contains spines (black arrowhead). F, large vacuoles in the parenchymal region (asterisk). G-H: G, Green nanoparticle-treated group: the normal-sized tubercles with blunt, short, reduced spines (black arrowheads). H, The tegumental area with a normal profile.

### Biochemical analysis

**Figure 5:**
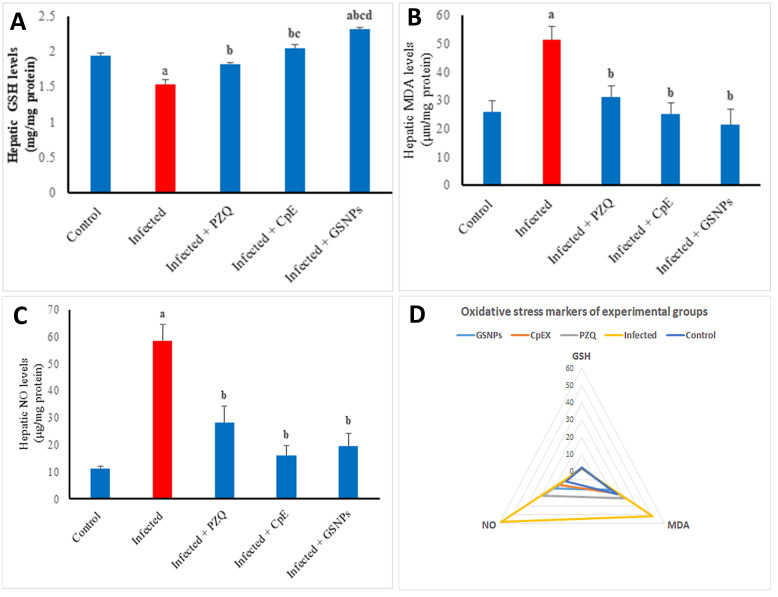
A&ndash;C: Effect of CpE, GSNPs, and PZQ on hepatic oxidative stress markers. A) Reduced GSH, B) MDA, C) NO, D) Antioxidant profile of experimental groups. Results are expressed as the Mean &plusmn; SEM. ^a, b, c, d^ are significant differences vs. the control, infected, PZQ, CpE, and GSNPs groups, respectively, at a p-value < 0.05.

The infection with *S. mansoni* led to a notable decrease in hepatic reduced GSH levels, showing a reduction of 21.27% when compared to the uninfected group (Fig. 5A). The administration of PZQ, CpE, and GSNPs led to a signifi-cant restoration of hepatic GSH levels, achieving increases of 19.19%, 34.33%, and 51.33%, respectively, when com-pared to the untreated infected group. The infection with *S. mansoni* resulted in a significant elevation of hepatic MDA and NO levels, with increases of 98.7% and 419.4%, respectively, compared to the uninfected group (Fig. 5B, C). The application of PZQ, CpE, and GSNP led to a notable decrease in oxidative stress markers. PZQ led to a reduc-tion in hepatic MDA and NO by 39.33% and 52%, respeс-tively, while CpE showed reductions of 50.98% and 72.8%, and GSNPs resulted in decreases of 58.66% and 66.4% when compared to the untreated infected group. Generally, it was found that the orally plant extract-treated group showed an antioxidant profile closer to that of the unin-fected group (Fig. 5D).

**Figure 6:**
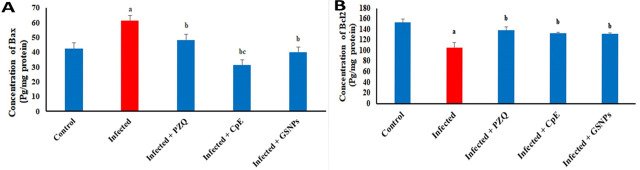
Effect of CpE, GSNPs, and PZQ on hepatic protein concentration of A) *Bax *and B) Bcl-2. Results are expressed as the Mean &plusmn; SEM. ^a, b, c, d^ are significant differences versus the control, infected, PZQ, CpE, and GSNPs groups, respectively, at a *p-value* < 0.05.

In terms of apoptotic markers, a significant rise in hepatic BAX concentration (44.4%) was observed in *S. mansoni*-infected mice when compared to the non-infected control group (Fig. 6A). Nonetheless, administration of PZQ, CpE, and GSNP resulted in significant decreases in hepatic BAX levels, with reductions of 21.4%, 48.9%, and 34.6%, respectively, when compared to the untreated infected group. Furthermore, the apoptotic pattern of the GSNP-treated group was found to be more similar to that of the uninfected group. The infected untreated group showed a decrease in the hepatic Bcl-2 concentra-tion (31.1%) in relation to the uninfected group (Fig. 6B). PZQ, CpE, and GSNP treatments demonstrated a remark-able recovery in the Bcl-2 levels (32%, 25.7%, and 24.9%, respectively) compared to the untreated infected group. The BAX/Bcl-2 ratio was more or less similar in the neg-ative control (0.277) as compared to the CpE- and GSNP-treated groups (0.236 and 0.304). The PZQ group revealed a higher ratio (0.347). This study suggests that the plant extract has therapeutic potential in promoting recovery from apoptosis.

**Figure 7:**
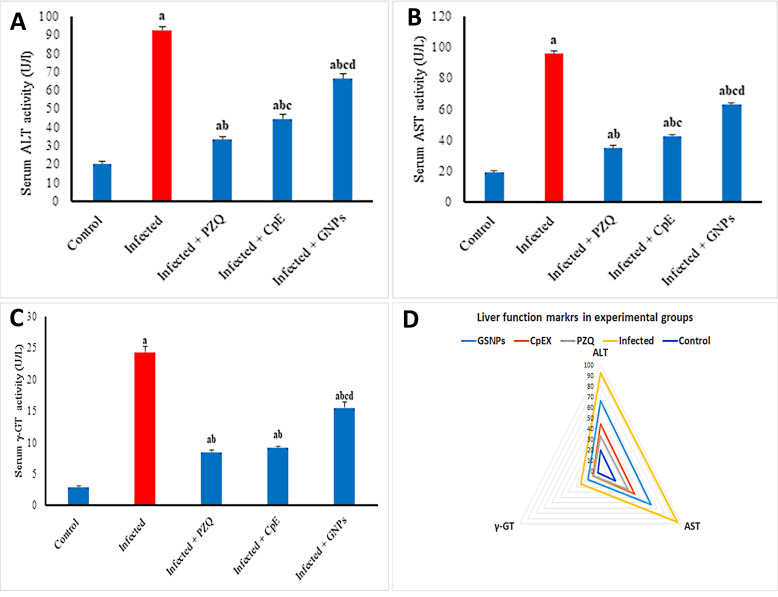
A&ndash;C Effect of CpE, GSNPs, and PZQ on serum A) ALT, B) AST, C) &gamma;-GT. D General profile of liver function markers in experimental groups. Results are expressed as the Mean &plusmn; SEM. ^a, b, c, d^ are significant differences versus the control, infected, PZQ, CpE, and GSNP groups, respectively, at a p-value < 0.05.

Infection with *S. mansoni* resulted in substantial increases in serum ALT, AST, and *y*-GT activity, with ele-vations of 368.35%, 409.33%, and 767.50%, respeс-tively, in comparison to the uninfected group (Fig. 7A-C). Pharmacological intervention with PZQ, CpE, or GSNPs sub-stantially reduced these elevations. PZQ specifically dimin-ished serum ALT, AST, and *y*-GT activity by 64.05%, 63.87%, and 65.18%, respectively. CpE diminished these enzyme activities by 52.16%, 55.45%, and 61.09%, whereas GSNP therapy decreased them by 28.38%, 34.30%, and 36.34%, respectively, compared to the untreated infected group. The PZQ-treated group had liver function profiles similar to those of the uninfected control group, followed by the CpE-treated group (Fig. 7D).

**Figure 8:**
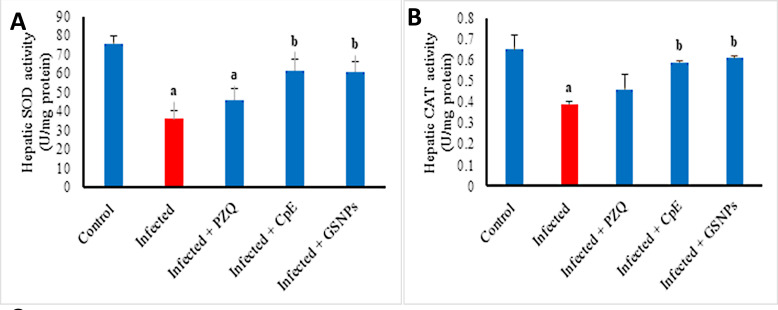
Effect of CpE, GSNPs, and PZQ on hepatic antioxidant enzyme activities. A) SOD and B) CAT. Results are expressed as the Mean &plusmn; SEM. a indicates a significant difference versus control, and b indicates a significant difference versus the infected group at a* p-value* < 0.05.

Infection with *S. mansoni* markedly reduced hepatic SOD and CAT activity by 52.28% and 40%, respectively, in comparison to the untreated group (Fig. 8A, B). The administration of PZQ resulted in a moderate 17.9% enhancement in CAT activity. Conversely, treatment with CpE and GSNPs led to significant enhancements in both CAT and SOD activity. CpE enhanced CAT and SOD activ-ities by 51.3% and 70.42%, respectively, whereas GSNPs augmented these activities by 56.6% and 68.24% com-pared to the untreated infected group.

**Figure 9:**
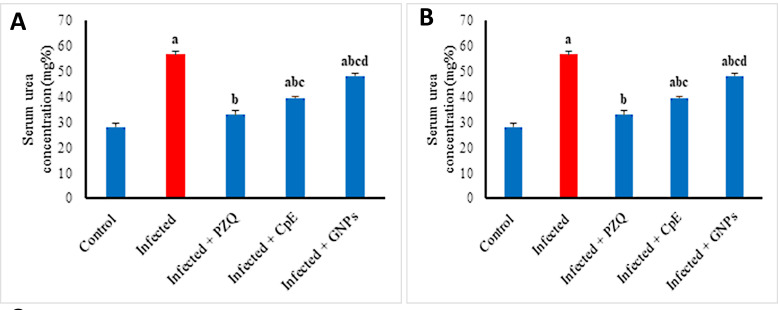
Effect of CpE, GSNPs, and PZQ on concentration of serum A) Urea and B) Creatinine. Results are expressed as the Mean &plusmn; SEM. ^a, b, c, d^ are significant differences versus the control, infected, PZQ, CpE, and GSNP groups, respectively, at a *p-value *< 0.05.

Serum urea and creatinine levels were significantly increased by *S. mansoni* infection, showing increases of 101.79% and 153.73%, respectively, compared to the uninfected group (Fig. 9A, B). The administration of PZQ, CpE, and GSNPs markedly diminished these increases. CpE diminished urea by 30.09% and creatinine by 41.18%, while PZQ lowered urea and creatinine levels to their mini-mum values of 42.05% and 42.35%, respectively. The GSNP treatment resulted in reductions in urea and creatinine lev-els, which decreased by 15.04% and 13.53%, respectively, compared to the untreated group. The PZQ-treated group demonstrated a kidney function profile similar to that of the uninfected group, followed by the CpE-treated group. All tables related to the above-mentioned data graphs of biochemical analysis are in Supplementary Tables 1-5.

**Figure 10:**
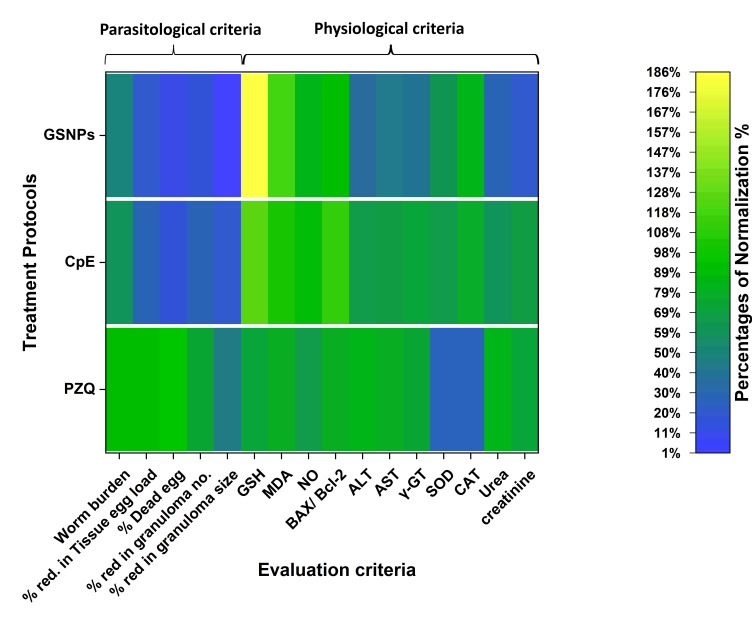
A heat map shows the normalization percentages in three therapeutic protocols applied in the Schistosomiasis infected murine system.

To objectively evaluate the three existing protocols for treating schistosomiasis, physiological criteria of the infected host must be considered in conjunction with par-asitological criteria. To achieve this evaluation, two meth-ods may be employed: either a visual evaluation using a heat map or a numerical evaluation using statistical meth-ods. First, all criteria, whether parasitic or physiological, must first be measurable accurately. Physiological criteria should be assessed in non-infected animals to establish standard values, which serve as a basis for calculating nor-malization percentages (or recovery ratios) in comparison to analogous physiological criteria in infected animals. This study established normalization percentages for the physi-ological criteria of infected mice (GSH, MDA, NO, BAX/Bcl-2, ALT, AST, *&gamma;*-GT, SOD, CAT, urea, and creatinine) and those related to the parasite (*Schistosoma*), including reduc-tions in worm burden, tissue egg load, granuloma num-ber, granuloma size, and percentage of dead eggs, within each treatment protocol separately, as presented in Table 5. All normalization percentages of this study were repre-sented in the visual graph "heat mapping" (Fig. 10), which shows that PZQ treatment is superior to other protocols.

Figure 5. A-C: Effect of CpE, GSNPs, and PZQ on hepatic oxidative stress markers. A) Reduced GSH, B) MDA, C) NO, D) Antioxidant profile of experimental groups. Results are expressed as the Mean &plusmn; SEM. a, b, c, d are significant differences vs. the control, infected, PZQ, CpE, and GSNPs groups, respectively, at a p-value < 0.05. Figure 6. Effect of CpE, GSNPs, and PZQ on hepatic protein concentration of A) Bax and B) Bcl-2. Results are expressed as the Mean &plusmn; SEM. a, b, c, d are significant differences versus the control, infected, PZQ, CpE, and GSNPs groups, respectively, at a p-value < 0.05. Figure 7. A-C Effect of CpE, GSNPs, and PZQ on serum A) ALT, B) AST, C) y-GT. D General profile of liver function markers in experimental groups. Results are expressed as the Mean &plusmn; SEM. a, b, c, d are significant differences versus the control, infected, PZQ, CpE, and GSNP groups, respectively, at a p-value < 0.05. Figure 8. Effect of CpE, GSNPs, and PZQ on hepatic antioxidant enzyme activities. A) SOD and B) CAT. Results are expressed as the Mean &plusmn; SEM. a indicates a significant difference versus control, and bindicates a significant difference versus the infected group at a p-value < 0.05. Figure 9. Effect of CpE, GSNPs, and PZQ on h on concentration of serum A) Urea and B) Creatinine. Results are expressed as the Mean &plusmn; SEM. a, b, c, d are significant differences versus the control, infected, PZQ, CpE, and GSNP groups, respectively, at a p-value < 0.05.

Regarding numerical evaluation, it was found that using the simple averaging statistical method [Arithmetic Mean (Equal Weight)] for all the present normalization percent-ages may be inaccurate because it mixes parasitological and physiological criteria, which are relatively independent, potentially masking strengths or weaknesses in each area. To address this, we calculated two separate efficacy scores (arithmetic mean of normalization percentage) for each protocol: one for parasitological criteria and one for physi-ological criteria. The equations used were as follows:


$$\text{Parasitological efficacy of protocol (PEP)}=\frac{1}{n_{p}}\sum_{j=1}^{n_{p}}X_{P_{j}}$$

where *n_f_* = number of parasitological criteria and *x_fi_* normalization percentage for parasitological criteria.


$$\text{Physiological Efficacy of protocol (PhEP)}:\frac{1}{n_{f}}\sum_{i=1}^{n_{inf}}x_{f_{i}}$$

where *n_f_* = number of physiological criteria and *x* = nor-malization percentage for physiological criteria.

A single numerical outcome for protocol evaluation can be created using a mathematical equation that combines parasitological and physiological efficacy scores. This technique can be customized based on biological priorities or statistical logic. The "Weighted Mean" is a statistical method that provides a comprehensive numerical value that reflects the overall effectiveness of the protocol across both measured PEP and PhEP criteria. It facilitates objec-tive comparison between protocols, even if their effects on individual parameters or criteria differ. "Weighted Mean" method depends on assigning weights to the values for which the mean is to be calculated.

In the present study, there are two sets of criteria (par-asitic and physiological) with two calculated values (PEP and PhEP) for each protocol (Table 6); to calculate the Weighted Mean of normalization percentage for each pro-tocol [Total Efficacy of Protocol (TEP)], the weights should be distributed according to the practical importance of each set. The present physiological criteria were identi-fied as clinically important and deserved 0.60 of the total weight, and the parasitological criteria were 0.40. TEP was calculated as follows:

TEP = PEP &times; its weight (0.4) + PhEP &times; its weight (0.6)

PEP = Arithmetic Mean of normalization percentage for a set of parasitological criteria

PhEP = Arithmetic Mean of normalization percentage for a set of physiological criteria

According to this statistical method and Tables 5 and 6, the Total Efficacies of the Protocols were 71.8%, 61.6%, and 51.1% for PZQ, CpE, and GSNPS, respectively (Table 6). Hence, this method considers the overall evalu-ation, not just the experiment's results but also the weight and importance of each measurement group in relation to the research objective. It was found that the effectiveness of the herbal treatment protocol was very close (despite its poor parasitic results) to that of PZQ. This is due to the herbal anti-apoptotic properties and its reducing effect on oxidative stress.

Figure 10. A heat map shows the normalization percentages in three therapeutic protocols applied in the Schistosomiasis-infected murine system.

Table 5

Table 6

## Discussion

This study aims to conduct a comprehensive objective evaluation of the use of three anti-schistosomiasis treat-ment protocols: natural extract (*C. procera*), nanomate-rial (GSNPs), and reference drug (PZQ). This evaluation is based not only on the anti-schistosomiasis effects, as is usual in most studies, but also includes the physiological responses of the host, especially the liver profile.

The extract of *C. procera* exhibited significant anti-schis-tosomal activity, primarily through its effects on *S. man-soni*. Research indicates that both alcoholic and aqueous extracts of this plant can reduce recovered worm and tis-sue egg load in infected mice, demonstrating its potential as a therapeutic agent against this neglected disease [7],[30]. Likewise, biogenic silver nanoparticles showed similar pharmacological effects against schistosomiasis. According to antiparasitic efficacy, it was found that the present ther-apeutic effect of the plant extract was stronger (in view of the worm burden, the egg tissue load, the oogram pattern, and the hepatic granuloma analysis) than that of the green nano-silver, in contrast to some other studies that showed that the effect of the green nano-silver was as close as to the strong effect of PZQ [5]. This difference can be explained by the fact that the present plant extract was given for a lon-ger period of up to 15 days, so the outcomes of some para-sitic parameters (worm burden and the size reductions of granulomas) were higher than those of a previous study [7], which applied *Calotropis* administration for only 5 days (60.4% and 19.3% *vs.* 46.4% and 11.22%, respectively). In addition, the larger size of the present GSNPs, reaching 108 &plusmn; 32 nm compared to 70-90 nm in Hamdan's study, causes a decrease in the therapeutic effect of the nanoparticles. It is worth noting that the present size variation among DLS and TEM data can be attributed to the fact that DLS measures the hydrodynamic diameter of particles in the solution, which includes the solvation shell and the green capping agents, while TEM measures the actual physical dimensions of dried particles under vacuum conditions [31]. The lack of accurate nanoparticle dose measurement using sophisticated analytical methods, such as inductively coupled plasma or graphite furnace atomic absorption spectrometry, is acknowledged as a drawback of our inves-tigation. This restriction resulted from the limited avail-ability of such equipment throughout the study period.

Regarding the tegumental modifications induced by the three treatments, it is known that these ultrastructural changes, resulting from the action of therapeutic agents, are one of the tools for diagnosing their therapeutic effi-cacy. Few studies have been conducted on the effect of sil-ver nanoparticles and CpE on the tegument of adult worms [5]. These tegumental changes included atrophy or col-lapse of the tubercles, sloughing, shortening of spines, and the formation of blebs. In the present TEM, similar variable alterations were observed in the worm tegument treated with either the silver nanoparticles or the plant extract. The plant extract-treated tegument showed an accumula-tion of mitochondria under the tegument in places where spicules were present. This finding may indicate the phar-macological effect of the extract compounds on the teg-umental areas in the spicule region, which may increase oxidative stress in these areas [32].

PZQ treatment produced the greatest reduction in both granuloma size and number, while *C. procera* extract showed moderate effects, and GSNPs had a limited but notable impact. However, CpE-treated mice exhibited the lowest fibrous content in granulomas, indicating a potential role in reducing fibrosis progression (antifibrotic effect), which is regarded as a significant finding for promoting a healthy liver profile [33]. The anti-fibrotic properties of the plant extract can be explained by considering several possible mechanisms. First, it significantly increases the activity of antioxidant enzymes (SOD and CAT), resulting in a reduction in the levels of oxidative stress markers (MDA and NO), as reported in the present study. This anti-oxidant effect helps neutralize both reactive oxygen spe-cies (ROS) and nitrogen species, which are involved in the activation of hepatic stellate cells (HSCs) and the progres-sion of hepatic fibrosis. Thus, the liver cells are protected from oxidative damage. Second, the extract's phytochemi-cals, including triterpenes such as Pomolic acid, inhibit the viability and activation of HSCs, the key cells responsible for extracellular matrix and collagen synthesis in fibrosis [34]. Third, CpE reduces pro-inflammatory cytokines (*e.g.*, interleukin-8) and, consequently, the inflammatory cell infiltration. The anti-inflammatory action helps decrease inflammatory stimuli that promote fibrosis through immune cell activation and HSC stimulation [35]. This was evident in the present histological sections of the liver in the extract-treated group, which showed a lack of immune infiltration around the central veins and portal areas, espe-cially when compared to those in the PZQ-treated group. Fourth, extract administration increased serum albumin levels, indicating improved liver synthetic function and hepatocyte regeneration [33]. Albumin acts as an anti-in-flammatory mediator by inhibiting TNF -a expression and NF-&kappa;B activation, thereby contributing to the resolution of fibrosis [36].

Likewise, GSNPs have shown a similar efficacy to that of the plant extract, considering the antifibrotic effect through the same possible mechanisms mentioned earlier. The studies demonstrated that silver nanoparticles inhib-ited the activation of HSCs and induced their apoptosis in a size- and dose-dependent manner [37]. Furthermore, the biosynthesized silver nanoparticles also downregulate matrix metalloproteinases (MMP-2 and MMP-9), which play a role in tissue remodeling during fibrosis [38],[39]. Besides, GSNPs significantly support antioxidant defenses by scavenging ROS [40],[41].

Regarding the liver enzymes, schistosomiasis resulted in the elevation of liver enzymes, including ALT, AST, and *y*-GT. Increased activity of ALT indicates liver injury, while increased activity of AST points to injury in peripheral tis-sues. *y*-GT is the most markedly increased enzyme, and because *y*-GT is associated with bile duct enzymes, this indicates a bile obstruction most likely due to a granuloma, which causes fibrosis and liver function impairment [42].

Considering the treatment protocols in this study, it was found that the liver enzymes (ALT, AST, and *y*-GT) were most improved after PZQ, followed by plant extracts, and although PZQ was not superior in increasing the antioxi-dant activity, it has this superiority in the normalization of liver enzymes [43],[44]. Several reported studies indi-cate that liver enzymes are improved with PZQ treatment due to the liver injury and fibrosis being addressed, as well as the PZQ's action mechanisms, which are similar to those previously described (belonging to plant extracts) [45]-[47]. Conversely, liver damage is improved with *C. pro-cera* extract, and inflammation is reduced, likely due to the extract's effect on the liver and fibrosis, as confirmed by evidence of increased serum markers [33]. This beneficial effect is likely due to *Calotropis* supporting the integrity of hepatocyte membranes and contributing to cell regenera-tion, thereby promoting normal levels of enzymes.

Regarding the hepatic apoptotic state, the *Schistosoma*-infected group showed disruption in the balance between pro-apoptotic and anti-apoptotic proteins (the BAX/Bcl-2 ratio), increasing BAX and decreasing Bcl-2, which shifts the liver environment toward apoptosis [46]. This mis-balancing occurs due to chronic inflammation caused by the infection, which first activates the NF-&kappa;В and JNK sig-naling pathways, leading to the upregulation of BAX and downregulation of Bcl-2 expression [47],[48]. Additionally, it increases ROS levels, which exacerbates mitochon-drial membrane damage and enhances BAX translocation [49],[50]. The BAX/Bcl-2 ratio is often considered a more informative prognostic marker than the individual expres-sion levels of BAX or Bcl-2 alone [51]. In the present study, this ratio was significantly closer (decreased) to the opti-mal values (negative control group) in the plant extract-and silver nanoparticle-treated groups. In contrast, a study stated that CpE increased BAX expression and reduced Bcl-2 levels in HepG2 cells [52]. This can be explained by the presence of a relative difference in the effective phy-tochemicals of the applied plant extracts, attributed to the type of solvent (water *vs.* methanolic), the part of the plant used (stems *vs.* roots), and species (*C. procera vs. C. gigantea*), respectively. Furthermore, a study [53] reported that calotropin, a bioactive cardenolide isolated from *Calotropis*, leads to the upregulation of BAX/Bak1, accom-panied by a significant reduction in Bcl-2 expression, in breast cancer patients. Generally, calotropin's pro-apop-totic effect, which involves increasing the BAX/Bcl-2 ratio, is well-documented in tumor models.

CpE has been shown to decrease apoptosis in liver cells of infected hosts, contributing to hepatoprotection. In *Plasmodium berghei*-infected mice, it was found that the treatment with *C. procera* leaf extract combined with bio-synthesized silver nanoparticles significantly reduced liver histopathological damage and oxidative stress caused by infection, suggesting decreased liver cell apoptosis [54]. This indicates that plant extracts (such as *Calotropis*) may have contradictory physiological effects depending on the nature of the organisms (infected animal, tumor envi-ronment, or even healthy animals). Accordingly, different physiological pathways may be induced by the same plant extract in organisms with different physiological environ-ments, or the plant extract may induce modifications in the dynamic equilibrium inside a specific physiological path-way. Regarding PZQ and hepatic apoptosis in this study, PZQ showed the lowest performance in normalizing apop-tosis levels. Many studies have reported that PZQ, in schis-tosomiasis-infected animals, normalizes liver pathology by reducing inflammatory and apoptotic responses [55].

It was found that the murine kidney function was affected by the schistosomiasis infection. That fact was represented by the significant elevations in serum urea and creatinine levels, indicating a renal dysfunction. This dysfunction was attributed to the elevated levels of inflammatory cytokines, such as TNF-a, IL-1*&szlig;*, and IL-6, in response to the formation of granulomas. These cytokines intensify the production of ROS in renal tissues, leading to tissue damage, which is followed by the accumulation of urea and creatinine in the bloodstream [56].

The present study showed that PZQ was the most effec-tive in reducing blood urea levels, followed by the plant extract. PZQ's effect on serum urea levels has been inves-tigated in infected animals, with generally minimal impact on urea concentrations. In *S. mansoni*-infected mice, PZQ treatment reduced kidney pathology, with some resto-ration of normal renal function biomarkers, including serum urea; however, changes were not always statisti-cally significant, depending on the dose and infection stage [57]. As in the results of the present study, CpE has been reported to affect serum urea/creatinine levels in infected organisms primarily through its nephroprotective and hepatoprotective actions, which help normalize kidney function disrupted by infection or toxicity [58],[59]. This nephroprotective effect is attributed to the antioxidant activity, which reduces oxidative stress in renal tissues, and anti-inflammatory properties, which mitigate tissue damage. It is worth noting that high doses or small-sized silver NPs may cause renal stress or toxicity, reflected by increased serum urea and creatinine and histopathological kidney changes [60]. In contrast, the present GSNPs have shown positive effects on serum urea and creatinine levels in animals infected with *Schistosoma*. At low or moderate doses, GSNPs generally do not cause significant changes in serum urea or creatinine in healthy animals [61].

From this study, it is clear that the plant extracts out-performed PZQ in improving liver efficiency by support-ing anti-fibrotic and antiapoptotic activities and reducing immune infiltration. The nanoparticles also exhibited the same anti-fibrotic effect on the liver; furthermore, they had the most significant improving effect on oxidative stress markers and antioxidant enzymes. On the other hand, although PZQ showed the highest antiparasitic effects (as indicated by the parasitic parameters), its physiological effects on the host were less pronounced in terms of oxi-dative stress markers, apoptosis, and antioxidant enzyme activities. Hence, it becomes clear that the actual evaluation of the three schistosomiasis protocols overlaps. Therefore, a comprehensive, objective evaluation of each protocol must be conducted by calculating the normalization percentages for each measured criterion, whether parasitic or host phys-iological, and then calculating the total efficacy of each pro-tocol according to the equations mentioned in this study.

## Conclusion

This study highlights the importance of assessing host liver and kidney function when evaluating antiparasitic treatments. *Calotropis procera* extract (200 mg/kg orally) and GSNPs (200 mg/kg intraperitoneally) both normalized the hepatic BAX/Bcl-2 ratio, indicating reduced apoptosis. Although GSH and MDA levels remained similar across treatments, the plant extract was more effective in lower-ing NO. Both treatments improved SOD and CAT activities, while PZQ and plant extract produced the greatest reduc-tions in ALT, AST, and *y*-GT. These findings support inte-grated evaluation of host physiology alongside parasitic endpoints. In general, determining the optimal treatment is challenging because each protocol involves variable factors, including nanoparticle size and vehicle, extract concentration and dose, and drug formulation. Regarding the solution to this problem, the present study estab-lished a mathematical technique that provides an overall evaluation of anti-schistosomiasis protocols, considering not only the experiment's results but also the weight and importance of each measurement criterion in relation to the research objective. Additionally, it is essential to con-duct multiple studies within each protocol separately, adjusting these variables to achieve a balanced therapeu-tic vision that considers both the more normalized physi-ological state of the host and the maximum anti-parasitic effect. In addition, future studies should integrate *in silico* approaches, including molecular docking and pharmacoki-netic modeling, to predict probable interactions between silver nanoparticles, plant phytochemicals, and parasite targets. This computational insight can guide nanoparticle design, optimize dosing regimens, and elucidate mecha-nistic pathways, thereby strengthening the translational potential of green silver therapies.

## Figures and Tables

**Table 1:** **Table 1.** The impact of treatments on the mean worm burden of various groups in comparison to the infected control group.

Groups	Male	Female	Couples	Total	Worm burden reduction (%)
Infected	1.8 &plusmn; 0.2	0.8 &plusmn; 0.20	3.4 &plusmn; 0.50	8.6 &plusmn; 1.07	
Infected + PZQ	0.00^a^	0^a^	0.4 &plusmn; 0.5^a^	0.8 &plusmn; 1.0^a^	90.6
Infected + CpE	0.6 &plusmn; 0.4^a^	0^a^	1.4 &plusmn; 0.24^a^	3.4 &plusmn; 0.4^a^	60.47
Infected + GSNPs	1.2 &plusmn; 0.2^b^	0^a^	1.6 &plusmn; 0.24^a^	5.0 &plusmn; 0.63^ab^	48.8

**Table 2:** **Table 2.** Oogram patterns in various treated groups versus the infected control one.

Groups	Immature ova	Mature ova	Dead ova
Infected	50.4 &plusmn; 0.50	44.8 &plusmn; 0.96	5.0 &plusmn; 0.31
Infected + PZQ	0 &plusmn; 0^a^	6.0 &plusmn; 0.44^a^	94.0 &plusmn; 0.44^a^
Infected + CpE	42.2 &plusmn; 1.49^ab^	41.4 &plusmn; 0.60^b^	16.4 &plusmn; 1.12^ab^
Infected + GSNPs	42.8 &plusmn; 1.15^ab^	45.4 &plusmn; 2.08^b^	11.8 &plusmn; 1.39^abc^

**Table 3:** **Table 3.** The mean number of ova per gm of liver and intestinal tissues in different treated groups versus infected control one.

Groups	Ova counts in liver (ova/mg tissue)	Ova counts in intestine (ova/mg tissue)	Total ova/mice	Reduction on ova count (%)
Infected	3,009.8 &plusmn; 320.31	2,916.4 &plusmn; 258.93	5,926.2 &plusmn; 471.76	
Infected + PZQ	227.2 &plusmn; 12.75^a^	203.8 &plusmn; 22.49^a^	431 &plusmn; 30.69^a^	92.73
Infected + CpE	1,917.4 &plusmn; 327.27^ab^	2,267.2 &plusmn; 63.22^b^	4,184.6 &plusmn; 366.27^ab^	29.39
Infected + GSNPs	2,249.2 &plusmn; 43.97^b^	2,548.2 &plusmn; 355.26^b^	4,797.4 &plusmn; 334.62^b^	19.05

**Table 4:** **Table 4.** The mean of numbers and sizes of hepatic granulomas in treated groups versus infected one.

Animal groups	Granuloma diameter (mean &plusmn; SE)	% Reduction in granuloma size	No of granuloma (mean &plusmn; SE)	% reduction (mean &plusmn; SE)	Types of granulomas			State of *S. mansoni* eggs	
					Cellular %	Fibro/ cellular%	Fibrous%	Intact	Degenerated
Infected	315.49 &plusmn; 15.73		10.6 &plusmn; 0.57		26 &plusmn; 3.61	74 &plusmn; 3.61	0	92 &plusmn; 1	8 &plusmn; 1
Infected + PZQ	169.18 &plusmn; 13.48^a^	46.3	2.67 &plusmn; 0.47^a^	74.8	1.67 &plusmn; 1.53^a^	96.33 &plusmn; 4.04^a^	0	17.33 &plusmn; 2.5^a^	82.67 &plusmn; 2.52^a^
Infected + CpE	254.04 &plusmn; 21.44^ab^	19.3	7.64 &plusmn; 0.86^ab^	28	21.33 &plusmn; 4.04^b^	86 &plusmn; 6.56	0	55 &plusmn; 3^ab^	45 &plusmn; 3^a^
Infected + GSNPs	312.32 &plusmn; 5.17^b^	1	8.8 &plusmn; 0.62^b^	16	25.67 &plusmn; 4.04^a^	74.33 &plusmn; 4.04	0	90 &plusmn; 1^bc^	10 &plusmn; 1

**Table 5:** **Table 5.** Normalization percentages of various experimental criteria of the whole study for evaluation of the total efficacies of the three treatment protocols.

Protocol	Parasitological criteria					The physiological criteria of the host												
	Worm burden	% red. in Tissue egg load	% Dead egg	% red. granu. no.	% red. granu. size	GSH	MDA	NO	BAX/ Bcl-2	ALT	AST	&gamma;-GT	SOD	CAT	Urea	Creatinine
PZQ	90.60%	92.70%	94%	74.80%	46.30%	79.20%	80%	81.40%	79.40%	90%	64.40%	73.60%	24.60%	25.40%	83.30%	69.40%
CpE	60.40%	29.30%	16%	28%	19.30%	125.60%	102.30%	90%	112%	66.30%	69%	70.10%	42.60%	41%	62.30%	82.60%	29.80%
GSNPs	48.80%	19.05%	11.80%	16%	1%	186%	118%	82.20%	93%	36%	36%	59.60%	64.30%	77.40%	67.50%	21.80%

**Table 6:** **Table 6.** Total efficacies of three treatment protocols according to "Weighted Mean".

Protocol	PEP %	PhEP %	TEP = PEP * (0.4) + PhEP * (0.6)
PZQ	79.68	66.55	31.872+39.93 = 71.8
CpE	30.68	82.23	12.27+49.33 = 61.6
GSNPS	19.32	72.30	7.72+43.38 = 51.1

## Data Availability

Not applicable

## Abbreviations

ALT, Alanine transaminase; AST, Aspartate transami nase; BAX, Bcl2-cell-associated X protein; Bcl-2, B-cell lymphoma protein 2; CAT, Catalase; CpE, Calotropis proc era extract; DLS, Dynamic light scattering; FTIR, Fourier transform infrared spectroscopy; GSH, Reduced gluta thione; GSNPs, Green-silver nanoparticles; &gamma;-GT, Gamma glutamyl transferase; JNK, c-Jun N-terminal Kinase; MDA, Malondialdehyde; NF-&kappa;B, Nuclear Factor kappa-light chain-enhancer of activated B cells; NPs, Nanoparticles; NO, Nitric oxide; PZQ, Praziquantel; SBSC, Schistosome Biology Supply Center; SOD, Superoxide dismutase; TWB, Total worm burden.
